# Fear of Death, Compassion, and Good Death among Portuguese Higher Education Students: an observational study

**DOI:** 10.1192/j.eurpsy.2026.11517

**Published:** 2026-07-31

**Authors:** C. Laranjeira, M. Pereira

**Affiliations:** 1 School of Health Sciences; 2 ciTechCare, Polytechnic University of Leiria, Leiria; 3 ULS Oeste–USF Rafael Bordalo Pinheiro, Caldas da Rainha, Portugal

## Abstract

**Introduction:**

Although discussing the inevitability of death involves everyone, previous evidence has focused more on stakeholders’ perspectives on the dying process and less on young, healthy adults.

**Objectives:**

This study sought to analyse the relationship between the concept of a good death, fear of death and self-compassion among higher education students.

**Methods:**

An e-survey was conducted in Portugal between November 2024 and January 2025 with 310 higher education students. The Good Death Concept Scale, the Collett-Lester Fear of Death Scale, and the Self-Compassion Scale were employed, along with a personal questionnaire. Descriptive analysis, independent sample t-tests, one-way ANOVA with post hoc analysis, and Pearson correlation analysis were all performed using JAMOVI statistical software (version 2.7.6). A multivariate linear regression analysis was used to determine the variables linked to death fear. The STROBE reporting checklist was followed in this study.

**Results:**

The mean age was 25 ± 8.52 years, with 75.2% of participants being female. The overall mean score for fear of death was 99.22 ± 21.97, signifying comparatively low levels of death fear. Health sciences students had elevated mortality anxiety levels in comparison to their non-health counterparts. Significant age and gender disparities were observed, with female and younger students exhibiting markedly elevated levels of death anxiety (p < 0.01). The Pearson correlation matrix revealed a positive link between fear of death and the concept of a good death, while demonstrating a negative correlation with self-compassion (p < 0.01). The main variables affecting the fear of death encompass age, gender, domains of closure and control, and the overidentification subscale (R2=0.352).

**Conclusions:**

Our results indicate that students frequently lack adequate preparation to confront death-related matters. Consequently, it is essential to implement curricular educational interventions centered on death education while actively engaging students in compassionate community efforts to enhance their awareness and self-assurance around end-of-life care.

**Disclosure of Interest:**

None Declared

